# Geographic Mosaic of Plant Evolution: Extrafloral Nectary Variation Mediated by Ant and Herbivore Assemblages

**DOI:** 10.1371/journal.pone.0123806

**Published:** 2015-04-17

**Authors:** Anselmo Nogueira, Pedro J. Rey, Julio M. Alcántara, Rodrigo M. Feitosa, Lúcia G. Lohmann

**Affiliations:** 1 Departamento de Botânica, Instituto de Biociências, Universidade de São Paulo, São Paulo, São Paulo, Brazil; 2 Departamento de Biología Animal, Biología Vegetal y Ecología, Facultad de Ciencias Experimentales, Universidad de Jaén, Jaén, Andalucia, Spain; 3 Departamento de Zoologia, Setor de Ciências Biológicas, Universidade Federal do Paraná, Curitiba, Paraná, Brazil; Indian Institute of Science, INDIA

## Abstract

Herbivory is an ecological process that is known to generate different patterns of selection on defensive plant traits across populations. Studies on this topic could greatly benefit from the general framework of the Geographic Mosaic Theory of Coevolution (GMT). Here, we hypothesize that herbivory represents a strong pressure for extrafloral nectary (EFN) bearing plants, with differences in herbivore and ant visitor assemblages leading to different evolutionary pressures among localities and ultimately to differences in EFN abundance and function. In this study, we investigate this hypothesis by analyzing 10 populations of *Anemopaegma album* (30 individuals per population) distributed through ca. 600 km of Neotropical savanna and covering most of the geographic range of this plant species. A common garden experiment revealed a phenotypic differentiation in EFN abundance, in which field and experimental plants showed a similar pattern of EFN variation among populations. We also did not find significant correlations between EFN traits and ant abundance, herbivory and plant performance across localities. Instead, a more complex pattern of ant–EFN variation, a geographic mosaic, emerged throughout the geographical range of *A*. *album*. We modeled the functional relationship between EFNs and ant traits across ant species and extended this phenotypic interface to characterize local situations of phenotypic matching and mismatching at the population level. Two distinct types of phenotypic matching emerged throughout populations: (1) a population with smaller ants (*Crematogaster crinosa)* matched with low abundance of EFNs; and (2) seven populations with bigger ants (*Camponotus* species) matched with higher EFN abundances. Three matched populations showed the highest plant performance and narrower variance of EFN abundance, representing potential plant evolutionary hotspots. Cases of mismatched and matched populations with the lowest performance were associated with abundant and highly detrimental herbivores. Our findings provide insights on the ecology and evolution of plant–ant guarding systems, and suggest new directions to research on facultative mutualistic interactions at wide geographic scales.

## Introduction

Variation in animal—plant interactions and the evolutionary divergence of these interactions among populations represent an important driver of morphological diversity [1,2]. Variation in the outcome of interactions often emerges from distinct geographic ranges between plants and animals in mutualisms [2,3] and antagonisms [4–9]; however, despite the importance of the geographic context, only recently this component was explicitly incorporated into ecological and evolutionary studies of interactions [2,10–12].

Ants can have positive (e.g., seed dispersal and plant defense) or negative (e.g., herbivory and seed predation) interactions with plants [13–15]. Ant—plant mutualisms are context-dependent *sensu* [16], especially in population-level comparisons due to local variation in biotic and abiotic conditions [17–21]. For example, the ant assemblages that visit EFNs or that disperse the seeds of a plant species vary across localities in terms of their species composition and function [20,22–25]. The outcome of these interactions is conditional on the composition of ant and herbivore assemblages and on the local abundance of alternative plant resources [7,8], what could drive differences across populations in herbivory intensity [22,26]. Whenever differences in the ant composition are translated into functional differences between assemblages, spatial and geographic variation in the ant visitor assemblages can lead to shifts in selective pressures on plant traits with potential for evolutionary divergence among localities [21,22,27,28].

Geographic variation in the outcome of animal—plant interactions has been considered a fundamental component of the co-evolutionary processes acting among interacting organisms. This idea lead to the proposition of the Geographic Mosaic Theory of Coevolution—GMT [10,11,29], which has been extended to the evolutionary outcomes of facultative plant—animal interactions [3,30]. This extension postulates that animal partners might influence plant evolution in some communities (i.e., plant evolutionary hotspots in which interactions lead to significant selection on plant traits) but not in others (i.e., plant evolutionary coldspots in which no significant selection mediated by animals occur due to different non-adaptive processes). Such pattern of geographic variation might lead to a mosaic across the plant geographic range in which the mean phenotypes of some plant populations are matched with the morpho-functional phenotypes of the assemblage of interacting animals, while other populations are mismatched [11] as described for an ant-plant seed dispersal mutualism [29, 31]. Admittedly, some of the phenotypic matching between plant and animal traits may arise by chance through neutral processes and not in response to the plant—animal interactions (giving rise to phenotypic match but evolutionary coldspot), but others are the result of adaptive processes resulting from the outcome of the interaction (phenotypically matched populations constituting evolutionary hotspots) [3, 31].

Here, we incorporate a geographic context into a study of interactions between a myrmecophile plant that bears extrafloral nectaries (EFNs) and the potential ant guarders visiting those EFNs. Herbivory can exert strong selection pressures on EFN—bearing plants, with differences in herbivore guilds and ant visitor assemblages among localities resulting in divergent selection on EFN traits and, eventually, leading to differences in the pattern and function of EFNs. Under this scenario standard evolutionary theory would predict a correlation across localities between herbivore damage and plant defensive traits (i.e., abundance of EFNs and nectar production) and ant guard services (i.e., number of ant visits or defensive attacks). Alternatively, under the GMT the evolution of plant defenses (EFNs) mediated by ants might proceed in a more complex way, not necessarily leading to a correlation between herbivory, EFN abundance, and ant guard defense. Under GMT scenario, we can expect a geographic mosaic with matched and mismatched populations; in matched populations, the phenotypes of EFNs are adjusted to the traits of the ant visitor assemblage, and this association could minimize herbivory conforming an evolutionary hotspot of the interaction; in contrast, in mismatched populations, EFN phenotypes are unrelated to ant traits and consequently to ant services (see Methods to operational definitions of matched and mismatched situations, as well as of evolutionary hotspot).

In this study we investigate ten populations of *Anemopaegma album* (Bignoniaceae) distributed across its geographical range and determine the relationship between quantitative EFN traits, morpho—functional properties of their associated ant assemblage and the outcomes of these interactions for the focal plant. More specifically, we address four main questions: (i) What is the pattern of geographical variation of EFN traits across populations of *A*. *album* (field and experimental plants)? (ii) How do ant visitors, herbivore assemblages, and herbivore damage vary across populations of *A*. *album*? (iii) Are the functional properties of each ant species (i.e., ant size and ant recruitment behavior) related to EFN traits, and if so, does this functional relationship translate into variation in plant defense and performance at the population level? (iv) Is the geographic pattern of interactions in agreement with the expectations of a geographic mosaic of plant evolution?

## Material and Methods

### Study system and plant populations

**Fig 1:**
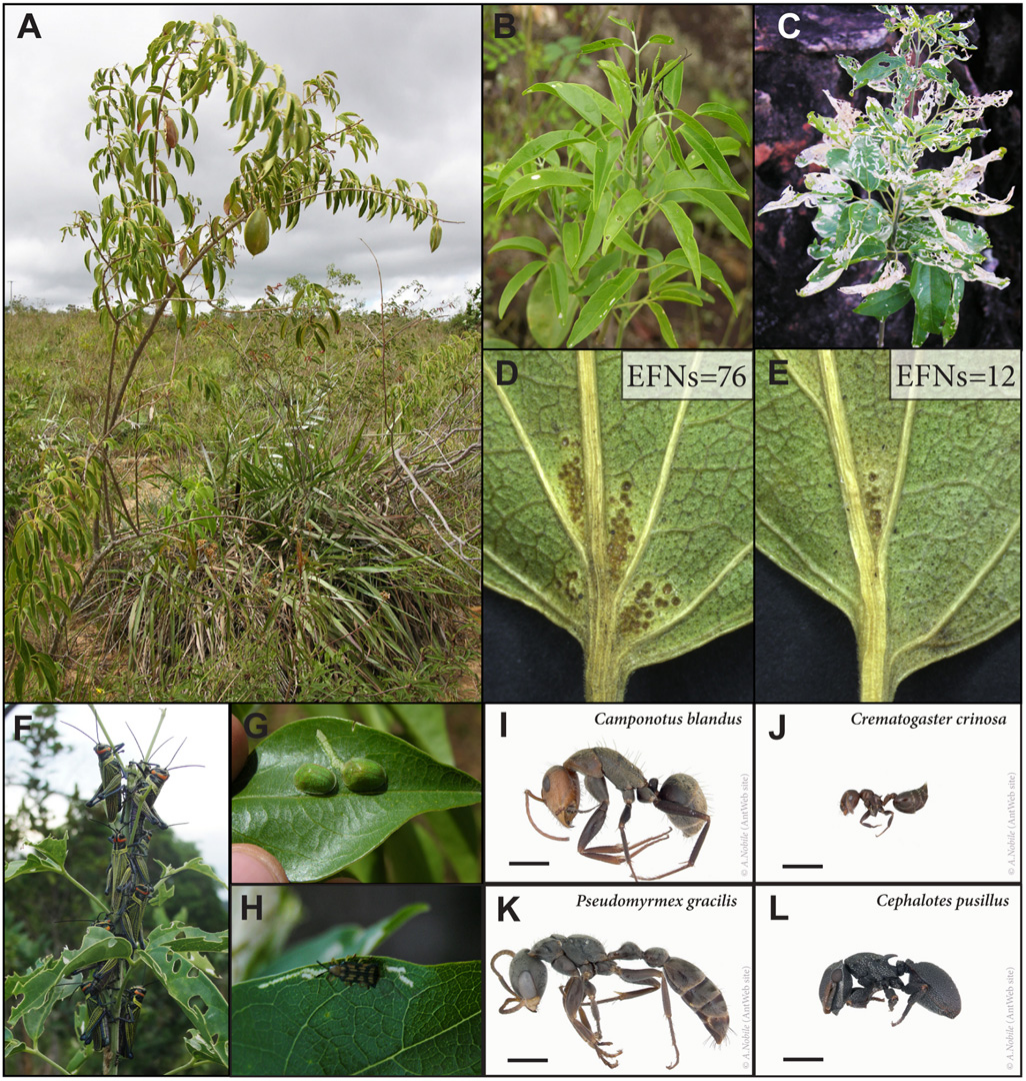
Ant—plant—herbivore system in a Neotropical savanna. A: Adult plant of *A*. *album* with immature fruits. B: Plant with nearly no leaf damage. C: Plant with severe leaf damage. D-E: Leaflets from different individuals of *A*. *album* showing wide variation in the number of EFNs on the base of leaflets (foliar cluster of EFNs). F-H: Most damaging herbivores for *A*. *album*: cricket *Xestotrachelus robustus*, and the beetles *Charidotis* sp. and *Sumitrosis* sp. I-L: Common ant species that visited EFNs, including four of the six most frequent ant species. Scale bars represent 1 mm in ant images; ant images are available in its original version in the AntWeb page (photo A. Nobile).

*Anemopaegma album* (Bignoniaceae) is a bee-pollinated and wind-dispersed [32] shrub (Fig 1A), with 3-foliolated leaves and large quantitative variation in extrafloral nectaries (EFNs) on leaflets. The EFNs are patteliform glandular trichomes that are mostly grouped at the base of the abaxial side of leaflets (Fig 1D–1E), and rarely sparsely distributed over the abaxial and adaxial leaflet blades. EFN abundance is positively related to ant visitor abundance [33]. At least three insect herbivores (Coleoptera and Orthoptera) feed on leaf tissues causing extreme foliage loss (Fig 1B–1C), while two caterpillars feed on flowers and fruits (A. Nogueira, pers.obs.).

**Fig 2:**
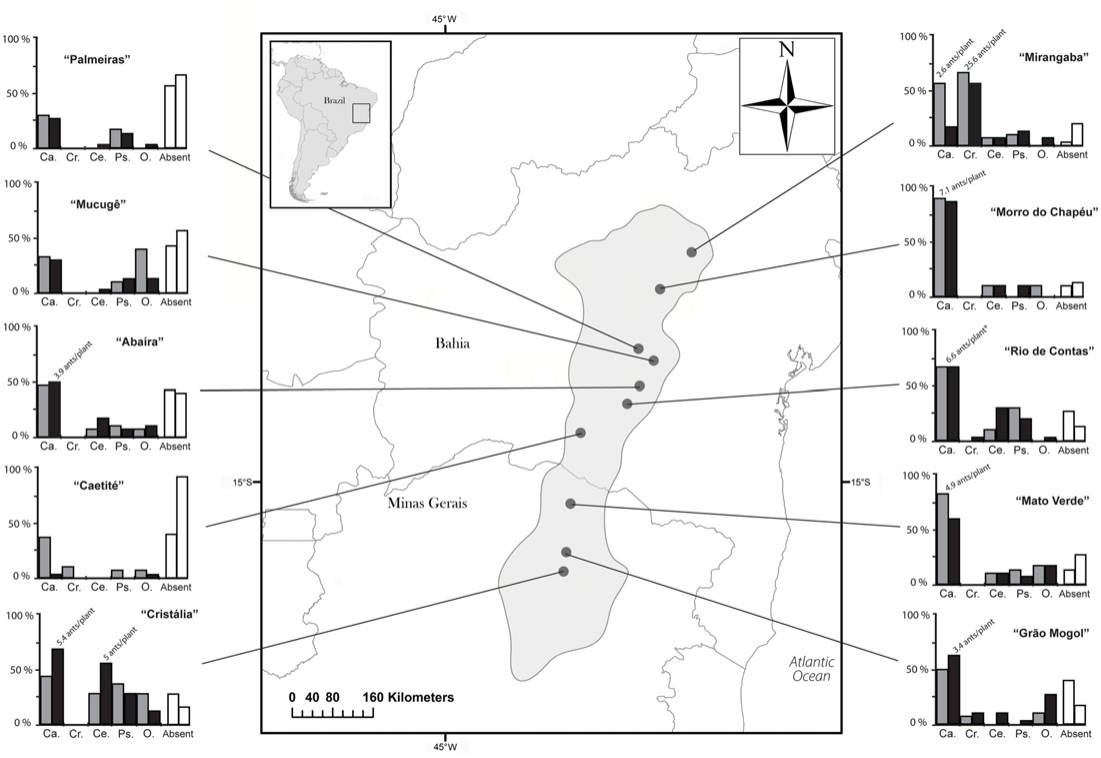
Map showing the geographic range of *Anemopaegma album* (gray) and the location of the ten experimental populations under study. Frequency graphs around the map show the occurrences of ant visitor assemblage at each population, depicted as the percentage of plants occupied by the most important ant genera that fed on EFNs: “Ca.” = *Camponotus*; “Cr.” = *Crematogaster*; “Ce.” = *Cephalotes*; “Ps.” = *Pseudomyrmex*; “O.” = others ant species (rare species); and “Absent” = proportion of plants without ants. Gray columns represent the frequency of ants at *t*_(0)_; and black columns represent the frequency of ants at *t*_(1)_. White columns represent the proportion of unoccupied plants in each population (*t*_(0)_ and *t*_(1)_ respectively, see Methods for additional details). The average number of ants per plant in each population is shown for the ant genera with more than 2.5 ants/plant in each population (the average ant abundance here is based on the absolute values without corrections based on plant size). In general, populations with the frequency chart on the right side were the populations with the highest ant visits.

Plants of *A*. *album* occur throughout the Brazilian states of Minas Gerais and Bahia (Fig 2), where they inhabit ‘cerrados’, ‘caatingas’, and transitional habitats known as ‘carrascos’. The climate is seasonal with temperatures ranging between 20.3° and 31°C in the rainy season and between 15.3° and 30.5°C in the dry season. The mean annual rainfall varies among localities between 650 mm in the north and 1.100 mm in the south, with accumulated mean rainfall in the rainy season around 710 mm (November–February) and around 40 mm (June–August) in the dry season.

We chose 10 sampling localities without evidence of human disturbance, preferentially within natural reserves, encompassing most of the geographic range of this plant species over 600 km of Neotropical savannas (Fig 2). All permissions to visit and collect biological dataset in Brazilian natural reserves were provided by the IEF-MG (authorizations number COL089/09 and COL090/08; ‘Parque Estadual de Grão Mogol’), SFC-DUC-BA (authorizations number NUBIO 03/2010; ‘Parque Estadual do Morro do Chapéu’), and SISBIO-ICMBio (authorization number 21979–1, ‘Parque Nacional da Chapada Diamantina’ and additional localities in MG and BA). In the cases carried out on private land, both the owner and SISBIO-ICMBio institution authorized the fieldwork proposed (authorization number 14505–4).

In each locality we systematically selected plants along three parallel transects, separated by 50 m from each other, planned to include specimens over the whole extension of each population. Thirty specimens of *A*. *album* were tagged avoiding plants distant less than seven meters from each other (S1 Table). Most populations were small, with less than fifty detected reproductive plants.

We surveyed each population in two sampling periods: (*t*_*0*_) October–November of 2009, at the onset of the rainy season when most individuals of *A*. *album* had produced new leaves; and (*t*_*1*_) March–April of 2010, at the end of the rainy season. At *t*_*0*_, plants were marked and geo-referenced, and the total number of leaves, flower buds and animal visitors—ant and herbivore assemblages—were sampled on each individual plant (detailed below). At *t*_*1*_, we collected the same data previously measured at *t*_*0*_, including ant and herbivore assemblages, and additionally counted the number of flowers per plant and the number of seedlings around each adult plant. At *t*_*1*_ we also collected 20% of the leaves of each plant to estimate herbivory levels and EFN traits, as well as fruits to perform a common garden experiment. Nectar secreted by EFNs of 15 plants at each population was also sampled at this time.

### Common garden experiment

In November 2010, seeds from fruits collected in the field were sown inside a greenhouse in the Bioscience Institute of the University of São Paulo (São Paulo, Brazil) under standard light availability, irrigation and edaphic conditions. We used PVC tubes with 1.20 m in height and 0.15 cm in diameter for seed planting, preventing radicular restriction during plant growth. Growing conditions included highly nutritional standardized soil (Tropstrato, Vida Verde), regular irrigation (twice/day) within the greenhouse (20 min each irrigation by micro sprinkler) and high irradiance (natural sun homogenized by a diffusing screen). We obtained a reasonable number of seedlings from different maternal plants in five populations, including Abaíra (N = 8 saplings/ 6 maternal plants), Cristália (N = 18 saplings/ 6 maternal plants), Palmeiras (N = 11 saplings/ 10 maternal plants), Grão Mogol (N = 12 saplings/ 8 maternal plants), and Mirangaba (N = 13 saplings/ 12 maternal plants). We collected leaves for EFN quantification in April 2013, after plants produced first flowers (i.e., plants that were two years old).

### Data collection

#### EFN traits

Extrafloral nectaries on leaflets of *A*. *album* (field and common garden plants) were quantified in the laboratory in 5–9 leaflets per plant using a stereomicroscope. To account for the distribution of EFNs in the leaflets, we estimated their abundance in three positions: (i) at the base of the abaxial surface of leaflets (1 cm along the central vein starting at the base of leaflet); (ii) along the abaxial surface of leaflets (1 cm^2^ in the middle portion of the leaflet and 1 cm^2^ next to the leaflet apex); and (iii) along the adaxial surface of leaflets (1 cm^2^ in the middle portion of the leaflet and 1 cm^2^ next to the leaflet apex). Given that on average 93% of EFNs clustered at the base of abaxial surface of leaflets, we used EFN abundance at this position as an overall measure of EFN abundance per leaflet. In addition, we measured the diameter of the secretory head of the largest nectary at the EFN cluster (base of abaxial side). We also collected samples of EFN secretion standardizing the number of hours to nectar accumulation and the number of leaves measured by individual plants. The bag exclusion procedure (with bridal veil bags) was prepared between 8:00 to 9:00 A.M, and nectar sampling was done at the same time in the following day, after the protected leaves accumulate nectar during a period of 24 hours. Nectar was sampled using 1μl and 5 μl microcaps, and quantified sugar concentration with a portable Eclipse refractometer (Standley, England).

#### Characterization of ant and herbivore assemblages

In *t*_*0*_ and *t*_*1*_ we sampled all ants and herbivores per plant in 15 min of visual censuses conducted between 7 A.M. and 11.30 A.M., and between 2.00 P.M and 6.30 P.M (herbivores that feed on *A*. *album* are mainly active in daytime), avoiding the highest temperatures at midday. Insect censuses were carried out during 20 consecutive days (two days in each locality) in each sampling period. We also observed ant and herbivore behaviors on plants to confirm the EFN use by ant species and the plant tissue damage by herbivores. Some specimens of ant and herbivore morphotypes were collected after the visual census and fixed in 90% alcohol for subsequent identification.

The relative frequency of plants occupied by ants, the average number of insects per plant and species composition were calculated in order to characterize the variation of ant and herbivore assemblages in each population. We used the number of leaves per plant as a measure of plant size. The number of ants visiting EFNs in each plant was standardized by the number of leaves, and re-scaled for plant size with an intermediate number of leaves (100 leaves). Therefore, the ant abundance per plant represent the average between the ant abundance (standardized) sampled at *t*_*0*_ and *t*_*1*_. Ant assemblage was also described using two functional features of each ant species: ant size and recruitment. Ant size was estimated by the mean total body length measured using a stereomicroscope on collected specimens (S2 Table). Ant recruitment was estimated as the maximum abundance of each ant species observed among all plants sampled (i.e., abundance standardized per plant size). We assume that the maximum abundance of each ant represents an intrinsic feature of each ant species. We used these measurements to calculate two functional traits for each population: the community-level ant size and the community-level ant recruitment; see [31] for a similar approach. These community-level traits were obtained as the average body size and recruitment of each ant species visiting EFNs weighted by the number of plants that the ants occupied in each population.

#### Herbivory and fitness

We collected 15–20% of leaflets per plant (range 12–58 leaves), systematically choosing one leaf from the apex, one from the middle portion, and one from the base of each branch (three leaves per branch). In the laboratory, we quantified total and damaged area on each leaflet by visual inspection with the aid of an acrylic sheet with 0.3 and 0.4 mm^2^ grids. Smaller grids were used to estimate the damaged area in leaves with smaller and scattered pattern of herbivory. Absolute herbivory per plant was estimated as the mean proportion of leaflet area damaged in each individual plant. Three performance descriptors were measured: (1) relative production of leaves, calculated as the difference in the number of leaves at *t*_*1*_ and *t*_*0*_ divided by the number of leaves in *t*_*0*_ (this descriptor varies from -1 to positive values); (2) proportion of flowering plants per population; and (3) number of seedlings (plants less than 50 cm in height) 2m-around each adult plant. We used the proportion of flowering plants per population because only the flowering plants could contribute to the positive fitness, independent of female and male components, in each locality. Additionally, the number of seedlings was used to represent the supra-annual cumulative (necessarily positive) fitness. Based on the seedling morphology (number of leaves and branches) and the variation between seedlings we suspect that these seedlings represent individuals from at least the last three years.

### Data analyses

#### Patterns of variation in EFN traits, ant visitor assemblages and herbivores

To characterize the similarity in ant and herbivore assemblage composition among localities (species level) we used the *Steinhaus* coefficient or proportional similarity index (*PS*). This index is the best known to be used with raw species abundances and compares two localities in terms of the minimum abundance of each species [34]. In this case, *PS* = 2*W*/(*A*+*B*), in which *W* is the sum of the minimum abundances of the various species, and *A* and *B* are the sum of the abundances of all species at each of the two sites (total number of specimens observed at each locality respectively) [34]. We tested the null hypothesis of “dissimilar assemblage composition” between localities (*PS* = 0; no taxa in common); following similar procedures as [23], and assessed the statistical significance for each coefficient of similarity by determining the 95% confidence limits through bootstrapping [35]. In addition, we explored whether the similarity of ant and herbivore assemblages, the population mean values of EFN traits, and the population performance depended on geographical distance. We built matrices of pairwise dissimilarity coefficients (1—PS) and geographical distances between populations. The dissimilarity measure applied to simple variables (e.g. total herbivore abundance) as the usual Euclidian distance. The spatial dependence of the ecological variables was tested using a Mantel test [34] with 5000 permutations.

#### Analyses of the functional links between ant and EFN traits, and its extension to detecting phenotypic matching and evolutionary hotspots

The phenotypic correspondence between EFN and ant traits, and between these traits and herbivory, were tested through linear regressions across ant-species. Two main questions were addressed: (i) Does the size or recruitment behavior of different ant species covary with the EFN traits of the plants they visit? and (ii) Does the size or recruitment behavior of ant species affect the levels of foliar herbivory? To answer these questions, we first obtained ant-specific EFN traits and herbivory levels as the average EFN traits and herbivory among all plants visited by each ant species. In general, ant censuses revealed only one ant species per plant; however, plants with more than one ant species on the EFNs (and its EFN phenotype) were considered for the characterization of the EFN abundance of plants that each ant species visit. For these calculations we only included ant species that visited more than five plants totalizing 15 ant species. We then used simple linear regressions to assess the bivariate relationships between EFN traits and herbivory versus ant size and recruitment across ant species (N = 15 ant species). We hypothesized a negative relationship between ant size and recruitment [36], and a positive relationship between ant size and EFN abundance due to the higher energetic requirement of bigger ant species (and consequently inverse relationship with ant recruitment). Finally, we considered these relationships as the phenotype-functional links (or phenotypic interface) between ants and EFNs. We used jackknife procedures in these regressions to avoid the effect of extreme values and applied one or two tail regression tests depending on the hypothesis previously formulated.

The phenotype-function relationships across ant species were subsequently used as an explicit model to identify situations of local phenotypic matching between the population average EFN traits and community-level ant traits. Specifically, we searched for populations in which EFN traits agree with the traits of their local ant assemblages (community-level ant size and/or recruitment) as it would be expected from the phenotype-function relationship, classifying the populations as matched when EFNs fit functional ant traits, and mismatched otherwise. This procedure was similar to that applied in the study of coevolutionary arms race between newts and snakes across populations, in which an explicit model was used to classify populations into phenotypic matched and mismatched [29,37].

Linear regressions were carried out between ant assemblage variables and the three plant population performance descriptors, in which we evaluated whether the defense provided by ants visiting EFNs was particularly effective in matched populations (i.e., they had lower level of herbivory and higher production of leaves, proportion of flowering plants and density of seedlings around mature plants). We also compared the variance of EFN abundance among populations using the Fligner-Killeen test (null hypothesis of equal variances), in which narrower variance of EFN traits was used as additional evidence that EFNs are suited to particular ant populations, in agreement with an adaptive scenario. In this way, populations phenotypically matched, with higher plant performance and lower variance of EFN abundance were considered as plant evolutionary hotspot, since these properties are expected under plant adaptation to the local ant visitor assemblages, and from a defensive hypothesis of resistance against herbivores mediated by ant guard.

The normality and homoscedasticity were checked in all linear regressions and normalizing transformation was applied in few cases [34]. Jackknife procedures were applied in some cases; see [38] for details on jackknife estimation. All statistical analyses were performed in R v. 3.0.1 [39] with standard and additional packages: bootstrap [40], geosphere [41] and Vegan [42].

## Results

### Patterns of EFN variation: natural populations and common garden experiment

**Fig 3:**
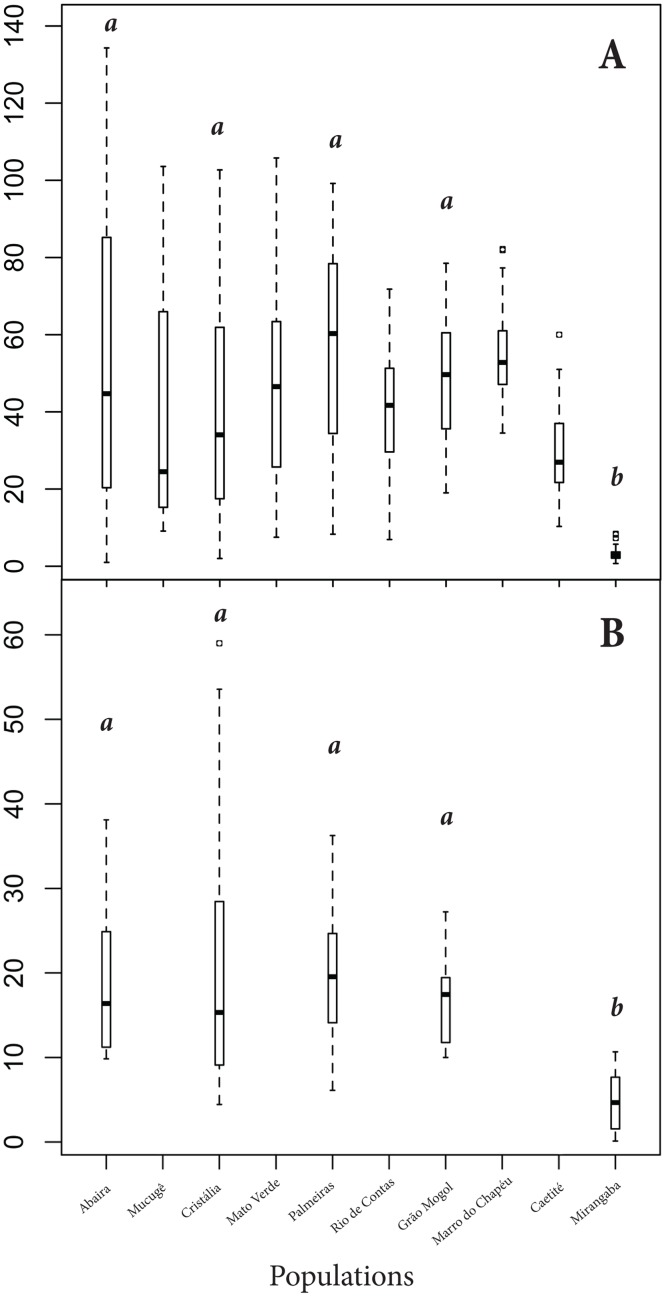
Variation of EFN abundance within and among populations of *Anemopaegma album* (Mean ± SD). A: EFN variation from field populations of *A*. *album* (N = 10 populations; F_(9,286)_ = 14.6; p≤0.001). B: EFN variation of plants grown in common garden (N = 5 populations; F_(4,58)_ = ; p≤0.001). In both cases, we detected differences in the abundance of EFNs among populations, in which Mirangaba had the smallest number of EFNs on the leaflets. Different letters indicate statistical differences in the Tukey post-hoc HSD test (p≤0.05).

All EFN descriptors varied significantly among field populations (EFN abundance: F_(9,286)_ = 14.59, p<0.001; and EFN size: F_(9,285)_ = 14.75, p<0.001). On average, plants from all populations had 41.4 ± 1.6 EFNs per leaflet (means are given ± 1S.E. unless otherwise stated) (S3 Table). The populations from Mirangaba and Caetité had the lowest EFN abundance (3.2 ± 0.4 and 29.6 ± 2.0 EFNs/leaflet, respectively), while Palmeiras and Morro do Chapéu had the highest values (56.7 ± 4.5 and 55.3 ± 2.3, respectively). EFN size varied among populations from 0.15 ± 0.002 mm (Mirangaba) to 0.20 ± 0.003 mm (Morro do Chapéu). The volume of nectar secreted varied from 0.01 to 5.41 μl/plant, and the sugar concentration from 13.7 to 55.6% (S3 Table). EFN traits (size, abundance, and nectar descriptors) did not correlate across populations (r<0.617; p>0.05; N = 10 in the three analyses). In addition, abundance and size of EFNs were not correlated with geographic distance (Mantel r: 0.230 and -0.004, respectively, p>0.17 in both cases). Although plants grown in common garden had fewer EFNs than plants in the field, the pattern of EFN variation in common garden (i.e., under controlled environment) was similar to EFN variation among field populations in the five populations compared (Fig 3).

### Proportion of plants being visited and the variation in ant visitor assemblage composition

We detected a large geographical variation in number of plants occupied by ants and average ant abundance per plant (Fig 2). The proportion of plants occupied by ants was positively correlated with the average number of ants per plant (r = 0.65; p<0.05; N = 10). The populations most visited by ants were Mirangaba (88% of the plants occupied with 5.5 ants/plant) and Morro do Chapéu (88% occupied with 5.6 ants/plant). In contrast, Caetité (33% occupied, with 3.1 ants/plant) and Palmeiras (38% occupied, with 0.6 ants/plant) were the least visited. Dissimilarity between populations in the abundance and proportion of plants occupied by ants were not correlated with geographic distance (Table 1).

**Table 1:** Mantel statistic and associated probabilities (p) for tests of general geographic dependency of all variables used to describe the ant—plant—herbivore interactions and fitness among populations.

	EFN traits		Ant descriptors					Herbivore descriptors			Fitness descriptors		
Statistics	Size (mm)	Abundance on leaflet base	Proportion of occupied plants	Abundance per plant	1-PS (Composition of ants)	Community level ant size	Community level ant recruitment	Total abundance	1-PS (Composition of herbivores)	Herbivore (%)	Relative production of leaves	Proportion of flowering plants	Density of saplings
r	-0.004	+0.230	-0.066	+0.100	+0.143	+0.044	**+0.342**	**+0.350**	+0.153	0.036	+0.060	+0.008	-0.002
p	0.49	0.17	0.60	0.23	0.21	0.35	**0.03**	**0.03**	0.17	0.40	0.38	0.36	0.49

Significant values are shown in bold (p≤0.05).

Populations with higher proportion of plants occupied by ants in t_0_ were also the most ant-occupied populations in t_1_ (Jackknife regression: b = 1.05 ± 0.73; and p = 0.006), evidencing the stability of the frequency of ant visits in each locality. Pooling all plants examined across populations (N = 300), 18% were not visited both in t_0_ and t_1_, while 52% were visited both times. Thus, 70% of plants showed stable occupancy by ants through the season. Among the plants that were visited by ants, 74% were consistently visited through the season by the same ant species. Morro do Chapéu and Mirangaba had the most stable ant visitor assemblage through the season (the ant visitor was the same in t_0_ and t_1_ in 90% and 83% of visited plants, respectively) and were the most stable in proportion of plants occupied by ants (S1 Fig).

Ant assemblage composition was relatively similar among populations in pairwise comparisons (average PS = 0.41 ± 0.02; S4 Table). The similarity in ant composition was due to the high abundances of *Camponotus* ants throughout *A*. *album* distribution and low abundances of *Pseudomyrmex* and *Cephalotes* (Fig 2). Ant visitor assemblages in Mirangaba and Cristália were quite different from the other populations. Mirangaba had a higher number of *Crematogaster crinosa* and Cristália a large number of *Cephalotes pusillus*. Dissimilarity of ant assemblage composition (1-PS_ants_) was also independent from geographic distance (Table 1).

### Functional features of the ant species feeding on EFNs

**Fig 4:**
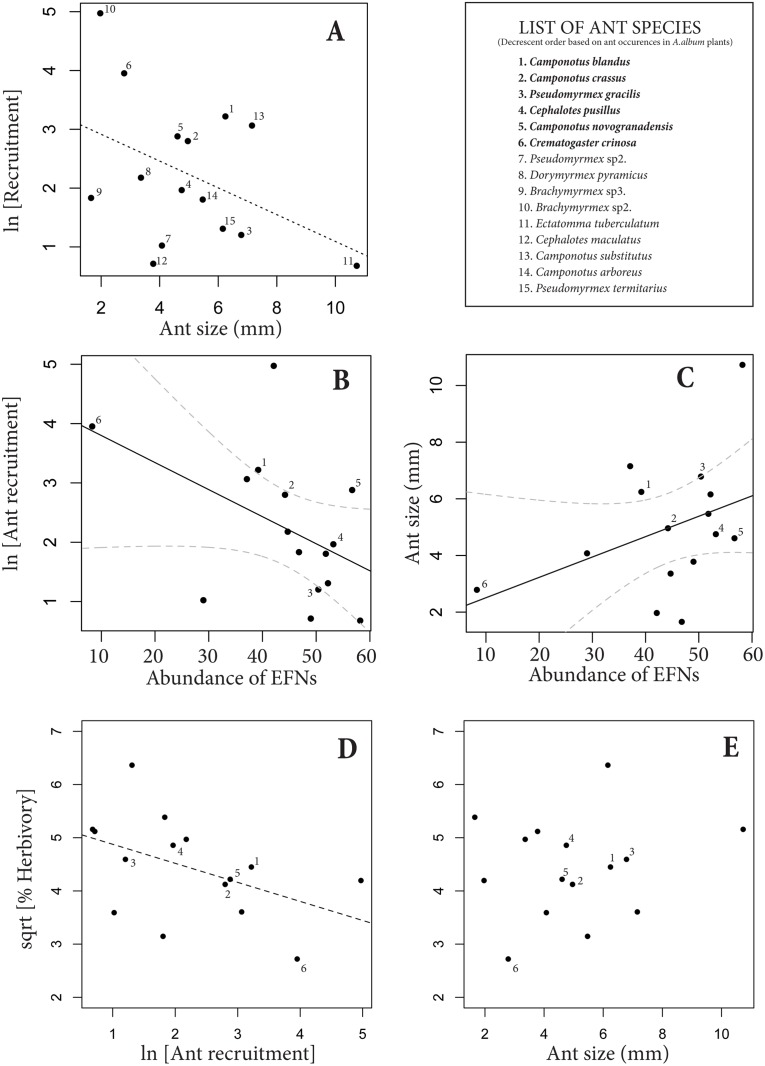
Relationship between ant traits (ant size and recruitment) and the average abundance of extrafloral nectaries (EFNs) and herbivory on plants visited by each ant species. Each point within the graphs represents one ant species (N = 15 ant species). The ant list is presented in decreasing order according to the ant occurrence on EFNs, highlighting the most important ant species in bold and numbered from 1 to 6 in the graphs. A: Smaller ant species generally had higher recruitment than bigger ants (F_(1,13)_ = 3.59; p = 0.06). B: Ant species with lower recruitment generally visited plants with higher amounts of EFNs (F_(1,13)_ = 3.06; p = 0.04). C: Smaller ant species generally visited plants with fewer EFNs (F_(1,13)_ = 2.38; p = 0.055). D: Plants that suffered less damage were generally visited by ants species with higher recruitment (F_(1,13)_ = 3.72; p = 0.06). E: Variation of ant size across ant species was not directly related to herbivory (F_(1,13)_ = 0.32; p = 0.58). All analyses are performed using jackknife procedures.

The four most frequent ant genera on EFNs had different behaviors (described in Nogueira *et al*. 2012a). *Crematogaster* was the most active recruiting, followed by *Camponotus* and *Cephalotes*, while *Pseudomyrmex* did not show a clear recruiting behavior. *Cephalotes* was quite passive in the presence of other insects on plants, while *Camponotus* were generally very agile, moving fast around nectar resources. Ant traits, EFN abundance and herbivory were functionally related. Smaller ant species generally had higher recruitment than bigger ants (Fig 4A). Ants with lower recruitment and bigger body sizes generally visited plants with higher amounts of nectaries (Fig 4B–4C); and ant recruitment, but not ant size, was negatively related to the average value of foliar herbivory across ant species (Fig 4D–4E), suggesting that the functional relationship between ants and EFNs effectively increases plant resistance against herbivores.

### Herbivores and herbivory

Populations with higher abundance of herbivores showed higher levels of foliar damage (Jackknife r = 0.62 ± 0.1; p<0.001; Table 2). Some of the herbivores observed in *A*. *album* actively fed on leaves and branches, while others were only detected through their traces on the plants (e.g., galling and some sucker insects). The most common and widespread herbivores were: *Charidotis* sp. (Chrysomelidae, Coleoptera), *Sumitrosis* sp. (Chrysomelidae, Coleoptera), and *Xestotrachelus robustus* (Romaleidae, Orthoptera) (Fig 1F–1H; S5 Table); these insects were responsible for the greatest proportion of leaf damage (AN observation).

**Table 2:** Pearson’s product-moment correlation between the mean population of EFN traits, ant descriptors (including functional features of ant community), herbivory and fitness descriptors.

	EFNs traits	Ant assemblage descriptors				Herbivore descriptor		Fitness descriptors		
Variables	Abundance on leaflet base	Proportion of occupied plants	Abundance per plant	Community- level ant size	Community- level ant recruitment	Total abundance of herbivores^1^	Herbivory (%)^1^	Relative production of leaves	Proportion of flowering plants^2^	Density of saplings
EFN size (mm)	+0.37 (±0.6)	-0.49 (±0.4)	-0.04 (±0.4)	+0.61 (±0.3)*	-0.02 (±0.7)	+0.09 (±0.4)	+0.50 (±0.5)	-0.38 (±0.5)	-0.10 (±0.5)	**-0.72 (±0.2)**
EFN abundance on leaflet base		-0.18 (±0.5)	-0.35 (±0.5)	+0.25 (±0.7)	-0.58 (±0.7)	-0.11 (±0.4)	-0.02 (±0.9)	+0.02 (±0.7)	-0.07 (±0.9)	-0.23 (±0.8)
Proportion of ant occupied plants			**+0.65 (±0.2)**	**-0.82 (±0.1)**	+0.18 (±0.4)	-0.33 (±0.3)	-0.62 (±0.3)	**+0.68 (±0.2)**	+0.47 (±0.3)	+0.60 (±0.3)*
Ant abundance per plant				**-0.65 (±0.2)**	**+0.66 (±0.2)**	-0.10 (±0.2)	-0.26 (±0.3)	**+0.64 (±0.3)**	**+0.59 (±0.2)**	+0.37 (±0.3)
Community-level ant size					-0.34 (±0.5)	+0.15 (±0.3)	+0.62 (±0.4)	**-0.81 (±0.1)**	**-0.64 (±0.2)**	-0.58 (±0.3)
Community-level ant recruitment						-0.20 (±0.3)	-0.30 (±0.6)	+0.40 (±0.4)	**+0.68 (±0.2)**	+0.37 (±0.5)
Total abundance of herbivores							**+0.62 (±0.1)**	-0.16 (±0.4)	-0.43 (±0.3)	-0.21 (±0.3)
Herbivory								**-0.74 (±0.3)**	**-0.76 (±0.1)**	**-0.73 (±0.2)**
Relative production of leaves									**+0.78 (±0.1)**	**+0.68 (±0.2)**
Proportion of flowering plants										+0.46 (±0.4)

All coefficients and probabilities were estimated by jackknife procedure and significant values are shown in bold (p≤0.05).

Asterisks (*) = p≤0.10;

and superscript numbers =

^1^square-root transformation;

^2^arcsine transformation.

On average, pair-wise similarity in the composition of the most common herbivores was smaller (average PS = 0.32 ± 0.03; S4 Table, below diagonal) than that observed in ant assemblages. Mirangaba and Morro do Chapéu had the lowest PS (statistically non-different from 0; S4 Table). Dissimilarity in herbivore composition (1—PS) was unrelated to geographic distance (Table 1). However, dissimilarity in total herbivore abundance increased with geographic distance (Mantel r = 0.350, p = 0.03; Table 1). In general, southern-most populations had greater abundance of herbivores than northern-most populations. When all populations were considered, herbivory damage was 30.2 ± 6.5%, with the smallest herbivory levels occurring in Mirangaba and Morro do Chapéu, and the highest in Caetité and Cristália (S5 Table).

### Detecting phenotypically matched and mismatched populations

**Fig 5:**
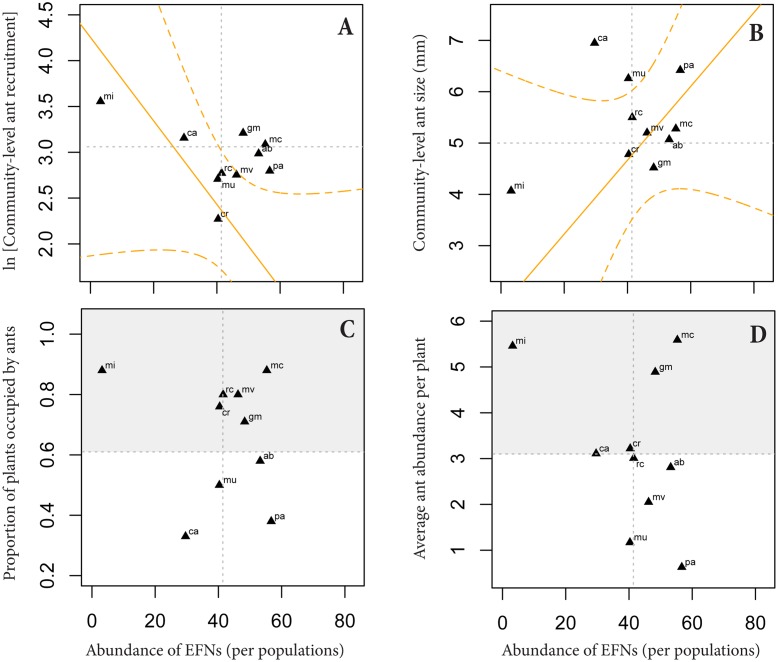
Matched and mismatched populations based on the local association between the community-level ant traits and the average values of EFN abundance. A-B: We used the phenotypic interface (or functional models) based on the relationship previously described between EFN abundance and ant traits across ant-species (Fig 4B–4C, and here highlighted in orange) in order to position the average values of EFNs and the community-level ant traits of each population. With this procedure, we were able to classify objectively each population in phenotypic matching and mismatching cases. Based on EFN abundance and community-level ant-recruitment (A) four populations were classified as mismatched populations. Based on EFN abundance and community-level ant-size (B) two populations, positioned outside the confidence interval (dashed orange lines), were classified as mismatched populations. C-D: The frequency of occupied plants (and the average ant abundance) was not clearly associated with the average EFN abundance, but confirmed the expected pattern of matched populations classified by community-level ant size and EFNs (evidenced by the gray region).

No clear correlation emerged across populations between ant functional traits and EFN abundance (three descriptors), size or nectar secretion (r < 0.61; N = 10; p<0.05 in all cases). More specifically, community-level ant size and recruitment were not correlated with EFN abundance at the base of leaflets (Fig 5A–5B). We also examined a more complex scenario of phenotypic matching and mismatching of *A*. *album* with their local ant visitor assemblages. We positioned each locality on the phenotype-function relationships between ant traits and EFN abundance (Fig 4B–4C; note that the regression lines and their confidence intervals in the Fig 5A–5B are the same depicted in Fig 4B–4C). Four populations were distant from the expected line depicted between EFN abundance and community-level ant recruitment (Fig 5A), and represent cases of phenotypic mismatching for this ant-plant trait combination. At least two of these populations, Grão Mogol and Morro do Chapéu, showed some of the highest values of ant abundance (4.9 and 5.6 ants/plant, respectively; Fig 5C–5D) and lowest herbivore damage (27.3 and 16.7%, respectively).

On the other hand, the phenotype-function relationships between EFN abundance and community-level ant size appeared as a better descriptor of matched and mismatched populations (Fig 5B). In this case, eight populations had the community-level ant size matched to the expected average values of EFN abundance based on the functional relationship depicted for these variables; only two populations, Caetité and Mucugê, appeared as mismatched (Fig 5B). Both mismatched populations were less visited by ants (3.1 and 1.1 ants/plant, respectively; Fig 5C–5D) and highly injured by herbivores (82.5 and 32.0%, respectively). Moreover, the eight matched populations were differentiated in two types of matching. The first type corresponded to Mirangaba, which was unique in showing low EFN abundance and small ants (lower left quadrant in Fig 5B). The second type of matching corresponded to other seven populations (Morro do Chapéu, Grão Mogol, Rio de Contas, Cristália, Mato Verde, Palmeiras and Abaíra), with higher abundances of EFN and bigger ant visitors (lower and upper right quadrant in Fig 5B).

### Describing plant evolutionary hotspots: phenotypically matched populations with elevated plant performance and low EFN variation

**Fig 6:**
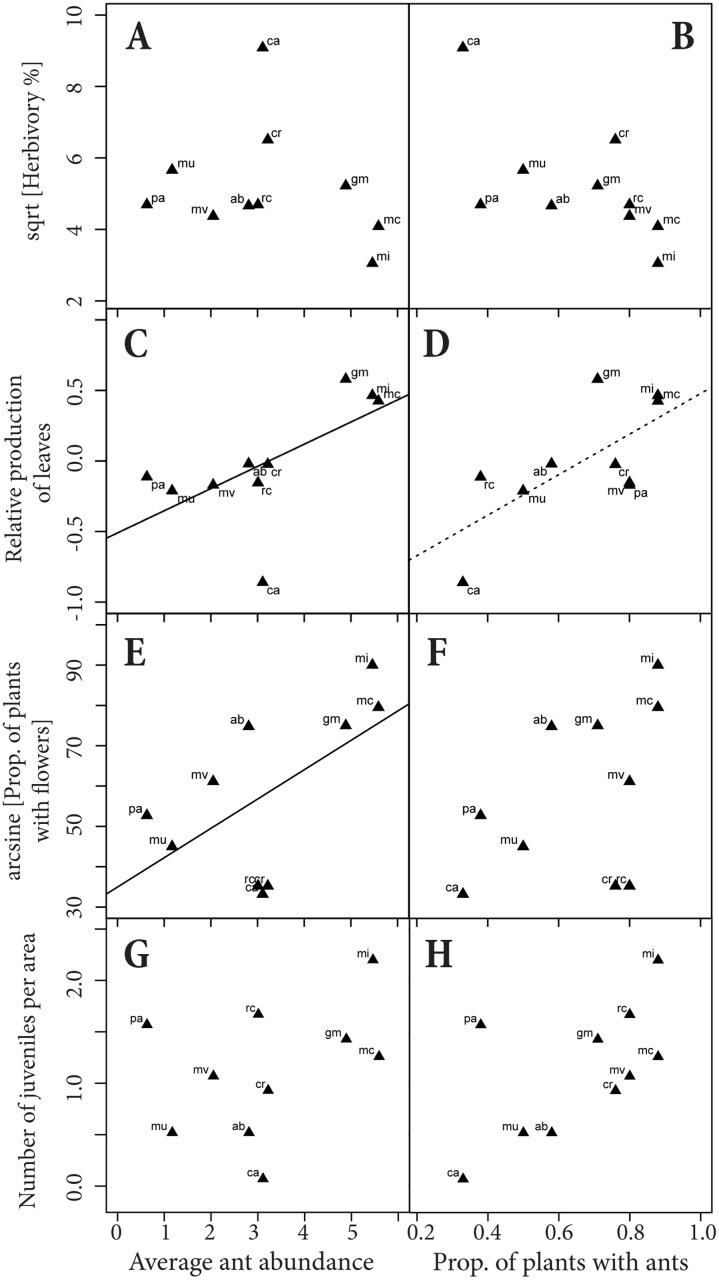
The average values of herbivory and three fitness descriptors per population were used to characterize the potential effect of ants on plants. Note the position of the three populations considered plant-evolutionary hotspots, in which the EFN—ant association could have a positive effect to the plants, decreasing herbivory and increasing fitness: Mirangaba (mi), Morro do Chapéu (mc) and Grão Mogol (gm). Solid and dashed lines had different levels of significance (p<0.05 and p<0.10, respectively).

There was no significant correlation among EFN traits and the descriptors of plant performance across populations (Table 2). In contrast, a significant relationship was found among average abundance of ants, some descriptors of plant performance, and community-level ant traits (Table 2 and Fig 6). In general, the higher the ant abundance, the higher the production of leaves and the proportion of flowering plants across populations (Fig 6C–6E). Populations with higher abundances of ants also showed smaller community-level ant size and higher community-level ant recruitment (Table 2). Moreover, the three descriptors of plant performance were also positively correlated with each other (Table 2).

**Fig 7:**
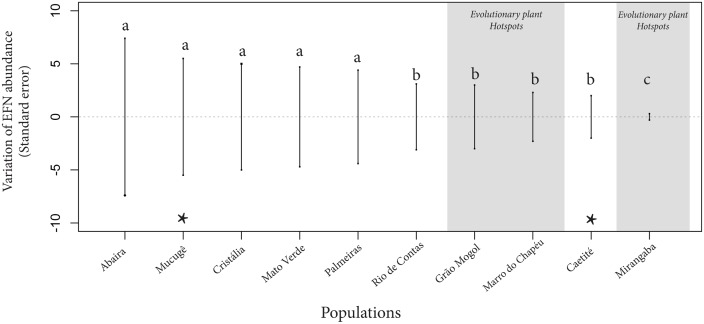
Standardized variation of EFN abundance within populations of *A*. *album*. Variance comparisons revealed two major groups with distinct patterns of EFN variance: (1) five populations on the left side with larger variances, and (2) the other four populations with narrower variances. The Mirangaba population appears differently from all other populations, with a narrower EFN variance. Three plant-evolutionary hotspots (matching populations with higher performance and narrower EFN variance) are highlighted in gray: Grão Mogol, Morro do Chapéu and Mirangaba. Asterisks indicate mismatched populations. Different letters indicate statistical differences among population variances (p≤0.05).

Few populations among those identified as phenotypically matched with their local ant assemblages had both high average values of plant performance and low variance of EFN abundance, and therefore are considered potential plant evolutionary hotspots (see Methods): Mirangaba, Morro do Chapéu, and to a lesser extent Grão Mogol. These three populations had higher values of ant abundance per plant (Fig 5D) and positive values of relative leaf production (Fig 6C–6D). In particular, Mirangaba was the population in which all descriptors of performance scored maximum values, with the minimum average values of herbivory, and the minimum variance of EFN abundance (Fig 7). Morro do Chapéu showed low levels of herbivory and high average values of at least two of the three performance descriptors (i.e., relative production of leaves and proportion of flowering plants). Grão Mogol showed a less clear pattern, with intermediate values of herbivory and high relative production of leaves (Fig 6). Grão Mogol and Morro do Chapéu had similar EFN variance that was greater than the variance on Mirangaba (Fig 7), but smaller than the other five populations. While the evolutionary hotspots Morro do Chapéu and Grão Mogol were driven by relatively large *Camponotus* ants, the Mirangaba hotspot was driven by small *Crematogaster* ants (Figs 2 and 5B).

## Discussion

This study represents the first attempt to analyze the evolutionary implications of an ant—plant—herbivore system mediated by extrafloral nectaries (EFNs) across a wide geographical scale; but see [22]. We argue that the geographic structure of these interactions in the myrmecochore shrub *A*. *album* (Bignoniaceae) agrees with the predictions of the Geographic Mosaic Theory—GMT [11]. We found a phenotype-function relationship that links EFN traits (abundance on the base of leaflets) and ant traits (ant species size and recruitment), which described the phenotypic interface of this interaction. Geographic variation in ant visits was positively related to plant defense and performance, but EFN traits did not covary across populations with ant abundance, herbivory or plant performance. We propose that the absence of such correlations with EFN traits could be an evidence of the existence of a geographic mosaic of local phenotypic matching and mismatching between EFNs and functional properties of ant assemblages, in which infrequent EFN evolutionary hotspots coexist geographically with frequent coldspots. The pattern of EFN variation in the field and common garden was quite similar, suggesting genetic differentiation of EFN abundance among populations. Such a mosaic seems to have been shaped not only by the ant—plant interactions, but also by the present-day ecological context in which the interaction happens at each locality, with bursts of herbivores that are not repelled by ants, conditioning the outcome of the interaction and temporarily cooling the evolutionary adjustment of EFNs to their local ant visitor assemblages.

### Patterns of EFN variation in *Anemopaegma album*

Despite the wide distribution and morphological diversity of EFNs, little is still known about the function of these secretory structures in most plant groups [43,44]. In general, larger EFNs, with more complex structures and vascular supplies (e.g., elevated EFNs in Leguminosae) secreted more nectar than non-vascularized glandular trichomes [45]. However, large numbers of small EFNs with lower structural costs, as the patelliform glandular trichomes in Bignoniaceae species (e.g., *A*. *album*), may be as effective as large vascularized secretory structures [45]. In the particular case of Bignoniaceae, the function of these small secretory structures has been corroborated in relation to its ability to attract ants [33,46], decrease herbivory [46,47], and increase plant performance [47].

In this study, we described considerable variation in EFN traits within and among populations. In ant—plant systems mediated by EFNs it would be expected that the variation of interactions would be largely determined by the variation in morphology/function of these secretory structures. For example, at the macroevolutionary level, the number of EFNs was positively correlated with the number of ant visitors in Bignonieae [48]. The same pattern was observed within a population of *A*. *album* [33]. However, the variation of EFN abundance was not clearly related to nectar descriptors and ant abundance across populations in our study. These discrepancies were caused by the ant visitor assemblage variation, with most populations being dominated by large *Camponotus* ants, and a single population dominated by the small *Crematogaster crinosa*. Different ant assemblages were associated with different EFN phenotypes, canceling-out the potential correlation across populations.

Very little is known about the genetic basis of EFN traits [49]. Some species shows significant genetic variation and heritability of EFN traits, including EFN size and proportion of leaves with EFNs [50]. The existence of genetic variation in EFN traits in wild populations provides a potential for adaptive responses [51]. In the specific case of Bignoniaceae, it has not been documented such heritability in any species, although preliminary analyses from quantitative genetic crosses in our common garden plants suggest that this is the case in some populations of *A*. *album* (Nogueira *et al*. unpublished). The evolutionary pattern of EFNs on Bignonieae [52] further suggests that they were heritable in the past and evolutionary labile due to adaptive processes [48].

### Variation in ant visitor assemblages

The four main ant genera that visited EFNs on *A*. *album—Camponotus*, *Crematogaster*, *Cephalotes* and *Pseudomyrmex*—are common in Neotropical savannas [53]. Nevertheless, individuals of *A*. *album* in most populations were predominantly visited by *Camponotus* (especially *C*. *blandus*, *C*. *crassus* and *C*. *novogranadensis*), an ant genus extensively documented for protection of plant tissues against herbivores in Neotropical savannas [54–57]. Furthermore, *Camponotus* species are known to be visually recognized by some herbivores; for example, ovipositing female butterflies select host plants without *Camponotus* ants in order to reach greater larval survival [58,59].

The importance of *Camponotus* throughout the distribution range of *A*. *album* may be due to the competitive hierarchy among ant species [60], with *Camponotus* outcompeting other ants. Alternatively, it is possible that *Camponotus* was strongly associated with specific conditions (e.g., plants with similar EFNs), leading to an apparent dominance over other ant species [61]. Nevertheless, *Camponotus* did not dominate the Mirangaba population, in which *Crematogaster crinosa* was the most abundant ant species. It is possible that its smaller body size may have favored the use of plants with fewer and less productive EFNs there. Indeed, ant body length was positively related with the quantity of sugar solution removed by workers among ant species [62]. Additionally, small ant body sizes are related to increased aggressive behavior, speed abilities and recruitment capacity [17,62–64].

### The association between EFN variation, ant and herbivore assemblages, herbivory and plant performance

We did not detect a clear association across populations between EFN traits and ant visitors, herbivore assemblages, intensity of herbivory, or plant performance. However, ant services (estimated by ant abundance and proportion of plants occupied by ants) explained the relative production of leaves and proportion of flowering plants. This pattern could reflect the balance between costs and benefits in ant—plant interactions [16,33,65], evidencing differences in the quality and quantity of ant services [66] with higher performance suggesting larger benefits than costs among populations, and leading a variety of possible outcomes on plants across localities [22,67]. In protection mutualisms, the costs and benefits may depend on the local abundance of the: (i) protector, (ii) beneficiary of protection, and (iii) natural enemy of the beneficiary [68]. Additionally, abiotic factors have been shown to also play important roles in ant—plant mutualisms; for example with *Inga vera* [20] and *Helleborus foetidus* myrmecochore system [18]. All of these components varied substantially across *A*. *album* populations reflecting the conditionality of these interactions; see [69] for context dependence.

Ant presence neither reduces herbivore damage nor increases plant fitness in two savanna species of *Anemopaegma* [33]. Likewise, shifts from tropical forests to savanna decreased EFN abundance in Bignonieae ancestors [48]. Together, these results suggest an apparent inefficiency of nectaries and its ants in the savannas that may reflect modifications of the ant and/or herbivore assemblages after the transition from forests. In this context, it would be expected that the EFNs of *A*. *album* would not be ‘efficient’ as a defensive trait in many populations, with its occurrence just resulting from phylogenetic inertia of EFNs during the evolutionary history of these plants [33]. The persistence of EFNs in *A*. *album* in spite of their relatively lower efficiency could be explained by a relative reduction of their cost. It can be argued that the cost of EFNs and its secretion would be low in savanna-like environments because the abundant light and accessibility to water (which is promoted by a deep root system) would provide large carbohydrate reserves to plants, allowing the maintenance of a relatively inefficient EFN, almost like a ‘neutral’ trait.

### The geographic mosaic of EFN evolution throughout the distribution range of *A*. *album*

The GMT extension to plant trait evolution in facultative plant—animal interactions hypothesizes the existence of mosaics of conspecific plant populations phenotypically matched or mismatched with phenotypic-functional attributes of their animal partner assemblages [29,37]. Such mosaics are established around a phenotypic interface (i.e., a phenotype-function relationship) between the plant and its animal partners. In this study, we detected such phenotype-function relationships between EFN abundance and ant species-specific size and recruitment. The transposition of the local population values of EFN abundance and ant assemblage traits into these phenotype-function relationships suggests that the set of studied populations comprises eight matched and two mismatched populations of *A*. *album*.

Phenotypic matching can arise through adaptive responses to selection pressures on EFNs exerted by efficient ant protection against local herbivores [22]. Alternatively, phenotypic matching can also arise through the dependence of EFNs and ant-assemblage traits on some common environmental factor. In this case, ant-assemblage traits would change by factors that are independent of *A*. *album* plants, with ants becoming temporarily matched with EFN traits. Indeed, the phenotypic matching can arise just by chance or stochastic factors (e.g., unknown reasons), without being adaptive. Chance could lead EFN traits to evolve through genetic drift and become temporarily matched with ant-assemblage traits in specific populations. Although our data does not allow us to rule out the possibility that individual populations are matched just by chance, the probability of finding one instance of matching by chance is small, while the probability of finding 8 out of 10 populations that are matched just by chance is vanishingly small.

Although neutral theory or chance might generate local situations of phenotypic matching, we would not expect higher average values of fitness or lower levels of EFN variance in those cases (both in accordance with an adaptive scenario of EFN evolution). In our study three of the eight matched populations (Mirangaba, Morro do Chapéu and Grão Mogol) had a high mean performance, minimum values of herbivore damage, and narrow variance in EFN abundance. These populations can be considered plant—EFN evolutionary hotspots, resulting from adaptive evolution, suggesting that a very efficient ant—guarding interaction is taking place in these localities. In contrast, matched populations that received high herbivore damage and achieved low mean performance should be considered plant evolutionary coldspots. Among the three hotspots, one population was from the southern extreme of the *A*. *album* distribution (Grão Mogol), while the other two were located in the northernmost localities, a discontinuous geographic distribution not related to spatial distance, illustrating the geographic mosaic nature of this interaction. A similar pattern was identified in a myrmecochore dispersal mutualism among localities [31].

We also identified a remarkable discrepancy in the nature of matching between the northern Mirangaba and Morro do Chapéu populations. The Mirangaba hotspot showed the smallest number of EFNs clustered at the base of leaflets (Fig 1E), and was matched with an ant assemblage dominated by small *Crematogaster* ants with a marked recruiting behavior. In contrast, the Morro do Chapéu hotspot had abundant EFNs clustered at the base of the leaflets, and was matched with an ant assemblage composed mainly by larger *Camponotus* ants with a lower recruiting behavior (Fig 1D). These results suggest that the mosaic can also diversify through divergent selection among hotspots; divergent selection is being explored in an ongoing study (A. Nogueira et al. in prep.).

Surprisingly, five out of the eight matched populations seem to be plant evolutionary coldspots: Palmeiras, Cristália, Rio de Contas, Mato Verde and Abaíra. These coldspots may have resulted from multiple non-adaptive processes. First, matched coldspots could have been hotspots previously, but their ant—EFN efficiency may have become temporarily disrupted by changes in the ecological settings (e.g., temporal abundance of other nectar resources for the ants, irruption of a particularly aggressive herbivore). Such disruptions are common in mutualistic interactions involving ants [70–72]. Gene flow from a nearby population could also contribute to the maintenance of phenotypic matching in a locality with an inefficient ant assemblage. The most evident coldspots of *A*. *album* were the mismatched populations of Caetité and Mucugê, where phenotypic mismatching was probably related to the scarcity of guarding ants (Fig 2) amounted with the higher abundance of large herbivores. Both populations were intensively injured by the biggest herbivore *Xestotrachelus robustus*, an aggressive cricket that feeds on mature and young leaves and branches (S5 Table, Fig 1F). Maintenance costs of EFNs in the absence of their benefits could result in counter selection on EFN traits [51], which would decrease their abundance and erode part of their genetic variation. This could be the case of Caetité, with the highest leaf area consumed and small number of ant visits, showing the lowest average EFN abundance (except by the Mirangaba) and an unexpectedly narrow EFN variance.

In sum, we provided evidence of a complex geographic structure of an ant guarding—plant—herbivore system that is congruent with expectations of the GMT of plant evolution. We also identified a phenotypic-functional link between ant and plant traits—ant size and EFN abundance—which might be related to the energetic requirements of ants, and could drive distinct evolutionary outcomes across the distribution range of *A*. *album*. The particular three-partner nature of this interaction [69] and the potential variability in time and space of the herbivore assemblage [73] seems essential in conditioning the optimization of ant—EFN defense on *A*. *album* plants in each locality. These findings constitute a first step in our research on the evolutionary ecology of this usual tropical interaction. Investigation linking EFN trait heritability, divergent phenotypic selection in relation to local ant and herbivore assemblages and population differentiation is still needed. However, our findings provide new insights into the evolution of plant—ant guarding systems at wide geographic scales.

## Supporting Information

S1 Fig Details of the ant dataset in the two sampling periods. (DOC)

S1 Table Details of studied populations of *A*. *album*. (DOC)

S2 Table Dataset of ant species visiting EFNs in *Anemopaegma album*. (DOC)

S3 Table Variation of EFN traits in 10 populations of *Anemopaegma album*. (DOC)

S4 Table Between-populations pairwise proportional similarity index of ant visitor and herbivore assemblages on *A*. *album*. (DOC)

S5 Table Herbivores responsible for the major damage in plants of *Anemopaegma album* in each population. (DOC)
