# Supplementary material for: Geographic Mosaic of Plant Evolution: Extrafloral Nectary Variation Mediated by Ant and Herbivore Assemblages
Source: PLoS One. 2015 Apr 17;10(4):e0123806. doi: 10.1371/journal.pone.0123806 (PMC4401756; doi:10.1371/journal.pone.0123806)

**Supplementary Material**

**S1 Figure:** Details of the ant dataset in two sampling periods (*t_0_* and *t_1_* separated three months). **A**: Populations with higher proportion of plants occupied by ants in *t_0_* were also the most ant-occupied populations in *t_1_*, evidencing the stability of the frequency of ant visits in each locality (Jackknife linear regression analyses with b = 1.05 ± 0.73; and *p* = 0.006; on the graph we illustrated the lowest smoother line using a locally weighted polynomial regression). **B:** Pooling all plants examined across populations, 18% were not visited both in t_0_ and t_1_, while 52% were visited both times. Thus 70% of plants showed stable occupancy by ants through the season. On the other hand, among the plants that were visited by ants, 74% were consistently visited through the season by the same species. **C**: Morro do Chapéu and Mirangaba (two hotspots of EFN evolution) had the most stable ant visitor assemblage through the season (the ant visitor was the same in t_0_ and t_1_ in 90% and 83% of visited plants, respectively) and were the most stable in proportion of plants occupied by ants. The higher temporal ant stability in these last populations could be an additional evidence for the evolutionary mosaic of ant-EFN evolution in *A. album* species.


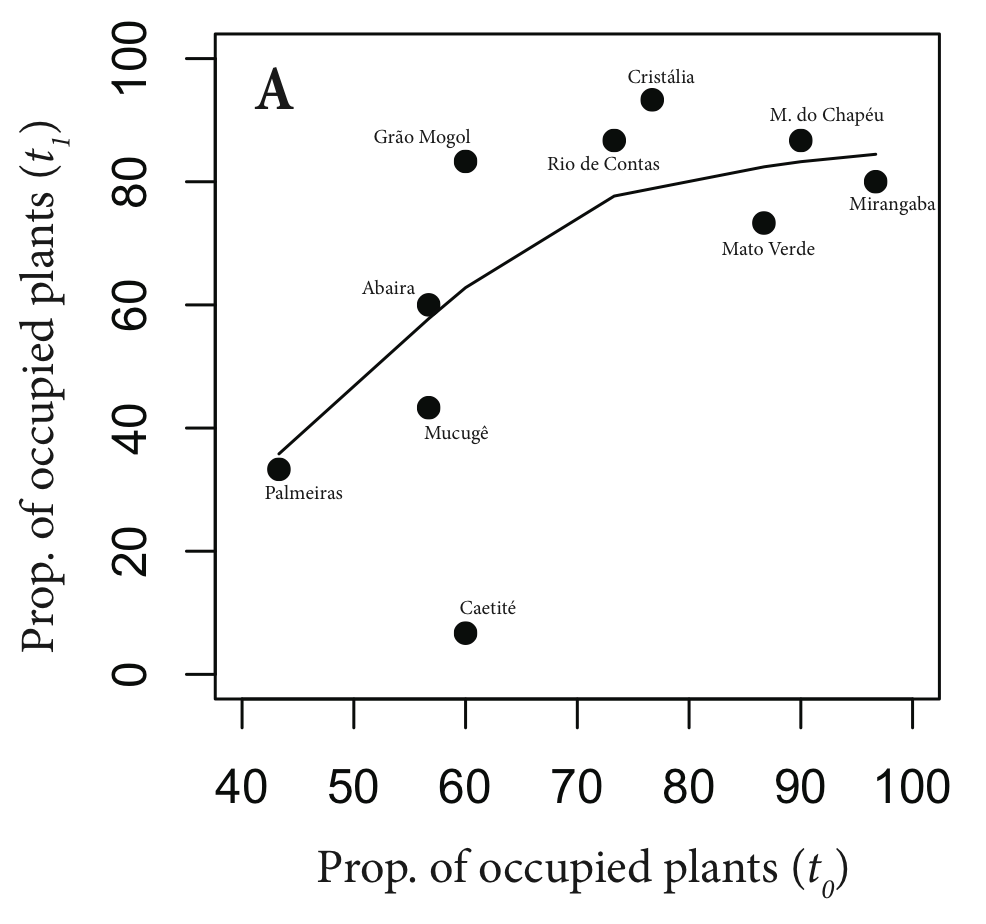

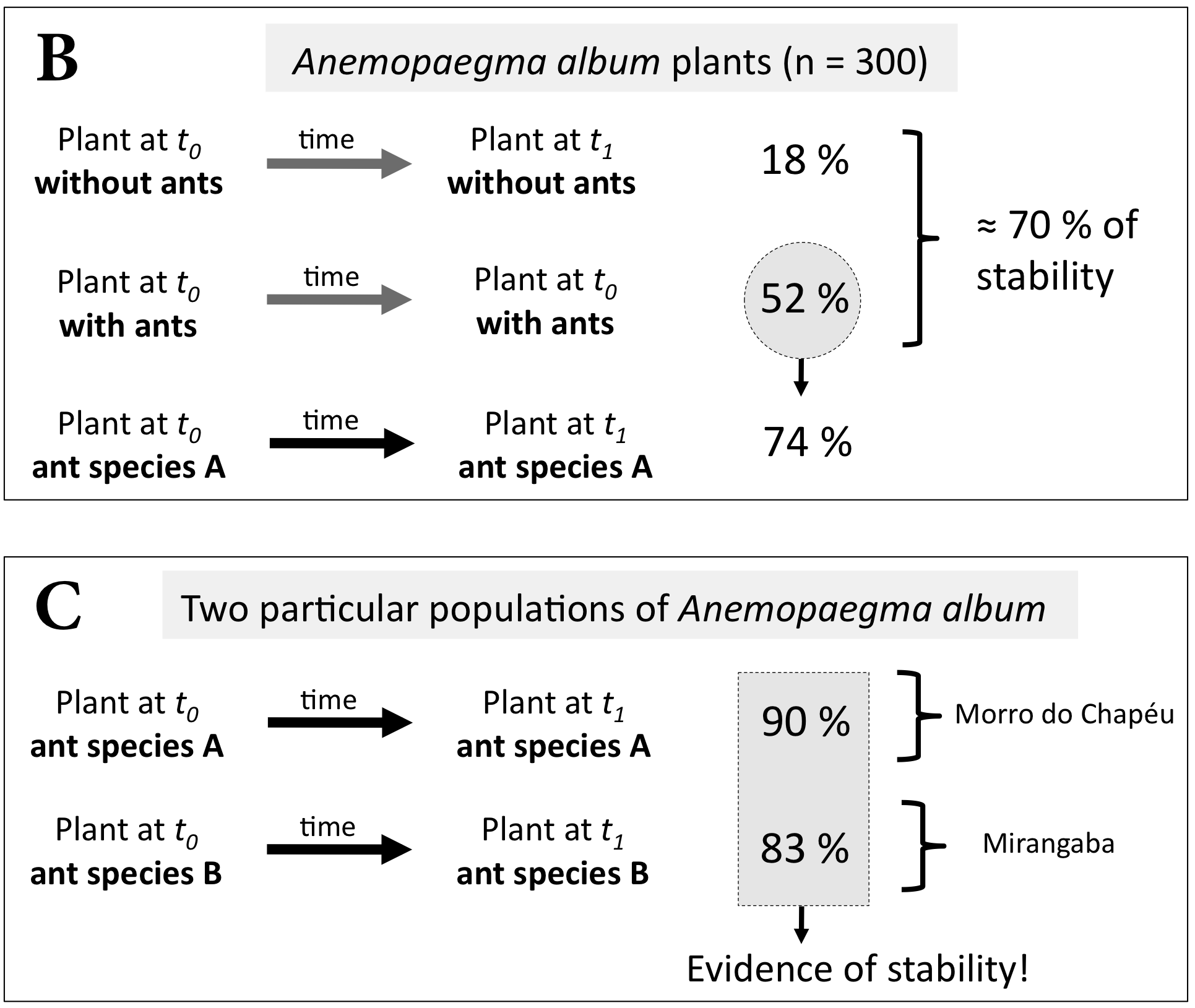

Supplement: S1 Fig — (DOC) [file pone.0123806.s001.doc]
