# Supplementary material for: Geographic Mosaic of Plant Evolution: Extrafloral Nectary Variation Mediated by Ant and Herbivore Assemblages
Source: PLoS One. 2015 Apr 17;10(4):e0123806. doi: 10.1371/journal.pone.0123806 (PMC4401756; doi:10.1371/journal.pone.0123806)
Supplement: S1 Table — (DOC) [file pone.0123806.s002.doc]

**Supplementary Material**

**S1 Table:** Details of studied populations of *A.album*. The minimum distance among individual plants within populations was three meters, but the majority of them were at least seven meters away. In each locality, we counted all plants visible along transects to estimate the total number of plants per population (this procedure is quite good to visualize plants in opened rocky areas within the savanna environment). Moreover, the idea here was not estimate well the total number of plants per population, but includes individuals along the whole plant population to estimate better the plant and insect descriptors throughout each population.

| Populations | The most distant plants (m) | Total area occupied by plants (m^2^) | Percentage of distant plants (≥ 7 m) (%) | Number of plants visualized in the field in each locality |
| --- | --- | --- | --- | --- |
| 1 - Abaira | 512 | 7432 | 87 | ≈ 57 |
| 2 - Caetité | 81 | 4583 | 80 | ≈ 45 |
| 3 - Cristália | 178 | 9337 | 90 | ≈ 118 |
| 4 - Grão Mogol | 280 | 35082 | 97 | ≈ 146 |
| 5 - Mato Verde | 135 | 4689 | 70 | ≈ 43 |
| 6 - Mirangaba | 93 | 4509 | 66 | ≈ 51 |
| 7 - Morro do Chapéu | 84 | 3988 | 63 | ≈ 35 |
| 8 - Mucugê | 683 | 38825 | 70 | ≈ 39 |
| 9 - Palmeiras | 203 | 9854 | 87 | ≈ 55 |
| 10 - Rio de Contas | 134 | 4201 | 53 | ≈ 33 |
| *Average* (n=10) | 238.3 | 12250 | 76.3 | 61.7 |
| **Average* (n=8) | 177.5 | 6074 | 74.5 | 54.6 |

**Average* = averages excluding the two populations with larger sampling areas (Grão Mogol and Mucugê).
