# Supplementary material for: Geographic Mosaic of Plant Evolution: Extrafloral Nectary Variation Mediated by Ant and Herbivore Assemblages
Source: PLoS One. 2015 Apr 17;10(4):e0123806. doi: 10.1371/journal.pone.0123806 (PMC4401756; doi:10.1371/journal.pone.0123806)
Supplement: S2 Table — (DOC) [file pone.0123806.s003.doc]

**Supplementary Material**

**S2 Table:** Dataset of ant species visiting extrafloral nectaries (EFNs) in 10 populations of *Anemopaegma album*. Shaded species were the most frequent ant species on EFNs. Occurrences on plants were based on the ant visits during two sampling periods (*t_0_* and *t_1_*), in which the ant dataset for each period was amounted here (per individual plant). Ant size was estimated as the average total length of individual workers.

| Ant species | Occurrences on plants | Recruitment (maximum abundance) | Ant size (mm) | *N* (ant size sampling) |
| --- | --- | --- | --- | --- |
| 1. *Camponotus blandus* | 96 | 10.0 | 6.24 | 86 |
| 2. *Camponotus crassus* | 95 | 16.4 | 4.96 | 99 |
| 3. *Pseudomyrmex gracilis* | 48 | 3.3 | 6.79 | 29 |
| 4. *Cephalotes pusillus* | 43 | 7.1 | 4.75 | 48 |
| 5. *Camponotus novogranadensis* | 27 | 17.8 | 4.61 | 27 |
| 6. *Crematogaster crinosa* | 25 | 52.0 | 2.79 | 23 |
| 7. *Pseudomyrmex* sp2 | 16 | 2.8 | 4.08 | 6 |
| 8. *Dorymyrmex pyramicus* | 15 | 8.8 | 3.36 | 24 |
| 9. *Brachymyrmex* sp3 | 12 | 6.2 | 1.66 | 7 |
| 10. *Brachymyrmex* sp2 | 11 | 144.4 | 1.97 | 8 |
| 11. *Ectatomma tuberculatum* | 11 | 2.0 | 10.73 | 9 |
| 12. *Cephalotes maculatus* | 9 | 2.0 | 3.78 | 7 |
| 13. *Camponotus substitutus* | 6 | 21.4 | 7.15 | 8 |
| 14. *Camponotus arboreus* | 5 | 6.1 | 5.47 | 6 |
| 15. *Pseudomyrmex termitarius* | 5 | 3.7 | 6.16 | 3 |
| 16. *Crematogaster obscurata* | 3 | 32.9 | 3.05 | 5 |
| 17. *Cephalotes pavonii* | 3 | 1.5 | 4.70 | 4 |
| 18. *Solenopsis* sp1 | 3 | 1.7 | 1.78 | 1 |
| 19. *Pheidole gertrudae* | 3 | 3.4 | 2.47 | 1 |
| 20. *Ectatomma edentatum* | 2 | 3.6 | 8.66 | 1 |
| 21. *Linepithema micans* | 2 | 0.8 | 1.78 | 1 |
| 22. *Camponotus rufipes* | 1 | 0.2 | 8.61 | 1 |
| 23. *Crematogaster* sp1 | 1 | 0.4 | 2.28 | 1 |
| 24. *Pseudomyrmex* sp1 | 1 | 0.9 | 3.46 | 1 |
| 25. *Pseudomyrmex* sp3 | 1 | 0.2 | 3.25 | 1 |
| 26. *Brachymyrmex* sp1 | 1 | 0.8 | 1.55 | 1 |
| 27. *Pheidole* sp1 | 1 | 1.5 | 1.75 | 1 |
