# Supplementary material for: Geographic Mosaic of Plant Evolution: Extrafloral Nectary Variation Mediated by Ant and Herbivore Assemblages
Source: PLoS One. 2015 Apr 17;10(4):e0123806. doi: 10.1371/journal.pone.0123806 (PMC4401756; doi:10.1371/journal.pone.0123806)
Supplement: S3 Table — (DOC) [file pone.0123806.s004.doc]

**Supplementary Material**

**S3 Table**: Variation of EFN traits (population average ± SE) in each of the 10 populations of *Anemopaegma album*. We did not find a clear positive correlation between the average abundance of EFNs and nectar (volume and/or concentration) across populations, but extreme differences were observed, with populations with few EFNs secreting smaller nectar quantities (e.g. Mirangaba).

| Populations sampled | EFN traits | | | | | |
| --- | --- | --- | --- | --- | --- | --- |
|  | Size* (mm) | Abundance (N= 27-32) | | | Nectar (N=15) | |
|  |  | Abaxial (base aggregated) | Abaxial (scattered) | Adaxial (scattered) | Volume | % Sugar |
| Abaira | 0.179 ± 0.005 | 53.2 ± 7.4 | 0.6 ± 0.22 | 0.9 ± 0.18 | 0.13 ± 0.08 | 55.6 ± 9.9 |
| Caetité | 0.203 ± 0.004 | 29.6 ± 2.0 | 0.3 ± 0.07 | 2.9 ± 0.38 | 0.74 ± 0.47 | 27.1 ± 5.9 |
| Cristália | 0.163 ± 0.004 | 40.4 ± 5.0 | 0.3 ± 0.06 | 0.9 ± 0.12 | 1.82 ± 0.64 | 26.6 ± 3.8 |
| Grão Mogol | 0.177 ± 0.004 | 48.3 ± 3.0 | 0.7 ± 0.20 | 0.8 ± 0.14 | 1.17 ± 0.43 | 46.6 ± 8.9 |
| Mato Verde | 0.175 ± 0.006 | 46.3 ± 4.7 | 2.0 ± 0.39 | 1.6 ± 0.18 | 4.67 ± 2.15 | 13.8 ± 1.9 |
| Mirangaba | 0.152 ± 0.002 | 3.2 ± 0.3 | 0.1 ± 0.01 | 0.2 ± 0.03 | 0.001 ± 2*10^-3^ | 45.6 ± 30.9 |
| Morro do Chapéu | 0.196 ± 0.003 | 55.3 ± 2.3 | 0.1 ± 0.01 | 0.3 ± 0.02 | 1.46 ± 0.52 | 24.3 ± 2.1 |
| Mucugê | 0.183 ± 0.003 | 40.3 ± 5.5 | 0.9 ± 0.30 | 0.3 ± 0.05 | 0.59 ± 0.29 | 35.5 ± 6.1 |
| Palmeiras | 0.173 ± 0.005 | 56.7 ± 4.4 | 1.4 ± 0.32 | 0.6 ± 0.05 | 1.06 ± 1.05 | 37.5 ± 16.0 |
| Rio de Contas | 0.159 ± 0.002 | 41.5 ± 3.1 | 1.1 ± 0.19 | 0.5 ± 0.05 | 5.41 ± 1.69 | 18.8 ± 2.3 |

Size* = diameter of the largest EFNs at the base of the abaxial surface of the leaflet blade (region of nectary clustering in the leaflets of *A. album*).
