# Supplementary material for: Geographic Mosaic of Plant Evolution: Extrafloral Nectary Variation Mediated by Ant and Herbivore Assemblages
Source: PLoS One. 2015 Apr 17;10(4):e0123806. doi: 10.1371/journal.pone.0123806 (PMC4401756; doi:10.1371/journal.pone.0123806)
Supplement: S4 Table — (DOC) [file pone.0123806.s005.doc]

**Supplementary Material**

**S4 Table: Pairwise proportional similarity index (PS) of ant visitor (above the diagonal) and herbivore (below the diagonal) assemblages on *A. album* calculated between populations. A bootstrap resampling procedure was used to assess if the PS-values are differed from zero (P ≤ 0.05), the maximum dissimilarity of species assemblages. PS-values different from zero are presented in bold.**

|  | Similarity | | | | | | | | | |
| --- | --- | --- | --- | --- | --- | --- | --- | --- | --- | --- |
| *Populations* | ABA | CAE | CRI | GMO | MVE | MIR | MCH | MUC | PAL | RCO |
| Abaira (ABA) | - | **0.28** | **0.51** | **0.34** | **0.59** | 0.18 | **0.61** | **0.66** | **0.27** | **0.65** |
| Caetité (CAE) | 0.07 | - | 0.17 | 0.35 | 0.37 | **0.38** | 0.34 | **0.32** | **0.54** | **0.42** |
| Cristália (CRI) | 0.03 | **0.59** | - | 0.27 | **0.52** | 0.22 | **0.38** | **0.38** | 0.21 | **0.64** |
| Grão Mogol (GMO) | 0.05 | **0.61** | **0.76** | - | **0.52** | 0.28 | **0.57** | **0.23** | 0.33 | **0.39** |
| Mato Verde (MVE) | **0.12** | **0.34** | **0.20** | **0.30** | - | **0.44** | **0.62** | **0.48** | 0.50 | **0.66** |
| Mirangaba (MIR) | 0.07 | 0.42 | 0.24 | 0.16 | 0.09 | - | 0.30 | 0.14 | 0.42 | **0.31** |
| Morro do Chapéu (MCH) | 0.10 | 0.58 | 0.27 | 0.15 | 0.07 | 0.64 | - | **0.45** | 0.41 | **0.63** |
| Mucugê (MUC) | **0.09** | **0.51** | **0.30** | **0.43** | **0.72** | **0.40** | 0.27 | - | 0.27 | **0.51** |
| Palmeiras (PAL) | 0.10 | **0.70** | **0.50** | **0.70** | **0.47** | 0.10 | 0.11 | **0.53** | - | **0.4** |
| Rio de Contas (RCO) | **0.19** | **0.25** | **0.14** | **0.21** | **0.75** | 0.11 | 0.06 | **0.56** | **0.35** | - |
