# Supplementary material for: Geographic Mosaic of Plant Evolution: Extrafloral Nectary Variation Mediated by Ant and Herbivore Assemblages
Source: PLoS One. 2015 Apr 17;10(4):e0123806. doi: 10.1371/journal.pone.0123806 (PMC4401756; doi:10.1371/journal.pone.0123806)
Supplement: S5 Table — (DOC) [file pone.0123806.s006.doc]

**Supplementary Material**

**S5 Table**: Herbivores responsible for major damage in plants of *Anemopaegma album* in each population. Herbivore abundance is described by the total number of organisms sampled from each population at *t_(0_*). Herbivore occupation is described as the number of plants occupied/total number of plants sampled (% in parentheses). Herbivory was estimated by the proportional amount of leaf tissue consumed. *Small beetle not identified that consumes very small portions of leaf tissue.

| Populations | Black beetle*  (Coleoptera) | Beetle *Charidotis* sp. (Coleoptera) | | | Beetle  *Sumitrosis* sp.  (Coleoptera) | Cricket *Xestotrachelus robustus* (Orthoptera) | Herbivory (%) | |
| --- | --- | --- | --- | --- | --- | --- | --- | --- |
|  |  | Adults | Larvae (variable instars) | Oviposition |  |  | Mean | SE |
| Abaíra | 14 (29%) | 1 (3%) | 5 (3%) | 2 (7%) | 0 (0) | 0 (0) | 21.8 | 6.0 |
| Caetité | 20 (43%) | 15 (17%) | 289 (17%) | 87 (40%) | 0 (0) | 278 (17%) | 82.5 | 5.9 |
| Cristália | 8 (26%) | 61 (57%) | 626 (53%) | 436 (90%) | 7 (10%) | 206 (17%) | 42.4 | 5.3 |
| Grão Mogol | 13 (27%) | 68 (53%) | 517 (63%) | 172 (90%) | 2 (3%) | 65 (7%) | 27.3 | 3.2 |
| Mato Verde | 1 (3%) | 8 (20%) | 109 (20%) | 46 (50%) | 0 (0) | 70 (13%) | 19.1 | 2.7 |
| Mirangaba | 1 (3%) | 2 (7%) | 0 (0) | 10 (13%) | 2 (7%) | 169 (17%) | 9.3 | 1.1 |
| Morro do Chapéu | 3 (7%) | 5 (17%) | 87 (27%) | 50 (70%) | 8 (23%) | 0 (0) | 16.7 | 1.5 |
| Mucugê | 18 (37%) | 7 (17%) | 1 (3%) | 1 (3%) | 53 (20%) | 284 (33%) | 32.0 | 3.7 |
| Palmeiras | 19 (37%) | 20 (33%) | 343 (30%) | 59 (53%) | 0 (0) | 20 (7%) | 22.0 | 2.4 |
| Rio de Contas | 3 (3%) | 9 (17%) | 57 (10%) | 27 (27%) | 2 (7%) | 1 (3%) | 26.6 | 3.8 |
